# An observational study on the safety of COVID-19 vaccination in patients with myasthenia gravis

**DOI:** 10.1007/s10072-023-06811-y

**Published:** 2023-05-09

**Authors:** H.Y. Wang, L. Qiu, C.Y. Ou, Z.Q. Lin, Z.D. Huang, P. Chen, Q. Ma, Y.R. Lu, H. Ran, W.B. Liu

**Affiliations:** 1 Department of Neurology, National Key Clinical Department and Key Discipline of Neurology, The First Affiliated Hospital, Sun Yat-sen University, Guangzhou, 510080 China; 2 School of Pharmaceutical Sciences, Sun Yat-Sen University, Guangzhou, 510006 China

**Keywords:** Myasthenia gravis, COVID-19 vaccine, Vaccination, Outcome

## Abstract

**Objective:**

There is concern that the coronavirus disease (COVID-19) vaccine may trigger or worsen autoimmune diseases. The objective of this study was to determine the impacts of COVID-19 vaccination on symptom severity in patients with myasthenia gravis (MG).

**Methods:**

A total of 106 enrolled patients with MG who were vaccinated against COVID-19 were followed up, and a questionnaire was used to document in detail the exacerbation of muscle weakness after vaccination and all other uncomfortable reactions after vaccination. Demographic, clinical characteristics, medication, and vaccination data were collected by follow-up interview. The main observation outcome was whether the MG symptoms of patients were exacerbated. The definition of exacerbation is according to the subjective feeling of the patient or a 2-point increase in daily life myasthenia gravis activity score relative to before vaccination, within 30 days after vaccination.

**Results:**

Of 106 enrolled patients [median age (SD) 41.0 years, 38 (35.8%) men, 53 (50.0%) with generalized MG, 74 (69.8%) positive for acetylcholine receptor antibody, and 21 (19.8%) with accompanying thymoma], muscle weakness symptoms were stable in 102 (96.2%) patients before vaccine inoculation. Muscle weakness worsened in 10 (9.4%) people after vaccination, of which 8 patients reported slight symptom worsening that resolved quickly (within a few days). Two (1.9%) of patients showed serious symptom aggravation that required hospitalization.

**Conclusion:**

Our results suggest that inactivated virus vaccines against COVID-19 may be safe for patients with MG whose condition is stable. Patients with generalized MG may be more likely to develop increased muscle weakness after vaccination.

**Supplementary Information:**

The online version contains supplementary material available at 10.1007/s10072-023-06811-y.

## Introduction

Coronavirus disease 2019 (COVID-19) is an acute infectious severe acute respiratory syndrome caused by the novel coronavirus SARS-CoV-2. Last 2 years, the COVID-19 pandemic has had a significant impact on global public health and social order. By 26 May 2022, there were over 526 million cases of COVID-19 worldwide, causing more than 6,290,170 deaths. The direct and indirect costs of controlling the spread of the disease are immeasurable. Despite the devotion of substantial efforts, evidence-based-specific therapies for COVID-19 remain scarce. In the absence of effective treatment, vaccination remains the most effective protective measure against COVID-19 infection.

Myasthenia gravis (MG) is an autoimmune disease [1]; the incidence of MG is approximately 20/million per year; the condition can occur at any age, and is the most frequent disorder of the neuromuscular transmission. MG is typically characterized by the production of antibodies against postsynaptic membrane acetylcholine receptor (AChR), followed by cascade transmission defects in muscle contraction, resulting in muscle weakness [2], which is relieved after rest and has fluctuating characteristics, with the extraocular, limb, and respiratory system muscles potentially affected. MG is treated by long-term administration of steroid and immunosuppressant drugs, and is characterized by frequent recurrence and a high incidence of complications [3]. Moreover, due to their pharmacologically induced immune-deficient status, superimposed with respiratory and/or skeletal muscle weakness, MG patients are both at a higher risk of COVID-19 infection and prone to consequent severe acute respiratory distress syndrome, deteriorating neurological symptoms, and higher mortality [4, 5].

To date, almost 300 COVID-19 vaccine candidates are being developed worldwide. So far, eight vaccines have been included in the WHO emergency use list and seven are licensed for use in China, including five inactivated SARS-CoV-2 vaccines, an adenovirus vector vaccine, and a recombinant subunit vaccine. Inactivated virus vaccine has been used for large-scale vaccination programs in China; as of November 28, 2022, more than 3.44 billion doses of COVID-19 vaccine were received nationwide, with more than 1.35 billion people vaccinated (data from the Joint Prevention and Control Agency of the State Council). Animal experiments and phase 1 and 2 clinical trials have consistently demonstrated that the inactivated SARS-CoV-2 vaccine has significant immunogenicity and low adverse reaction rates [6]; however, the rapid development of the COVID-19 vaccine has also led us to consider the safety and efficacy of the vaccine in immunocompromised patients, such as those with MG or who are taking immunosuppressant drugs, as these patients are often excluded from vaccine clinical trials. Whether vaccination increases the risk of deteriorating symptoms, or whether the use of immunosuppressants reduces the effect of the vaccine is unclear. Currently, there are no reliable data on the safety of the COVID-19 vaccine in patients with MG, nor are there informed guidance recommendations for vaccination of patients with MG during the COVID-19 pandemic. In our study, we followed up patients with MG patients in our center who were vaccinated against COVID-19 and reported their disease information and clinical course after vaccination, with the aim of assessing the safety of the COVID-19 vaccine in patients with MG, to provide guidance for vaccination recommendations.

## Methods

### Subjects

This was a retrospective, single-center, observational study. Of patients with MG (*n* = 1209) who were diagnosed in our hospital from January 2016 to October 2021, 106 patients were vaccinated against COVID-19 and were reviewed at outpatient or telephone (some patients were unable to go to the center during the outbreak) follow-up. The study was launched in October 2021 through outpatient surveys and telephone follow-up until April 2022. Diagnosis of MG was based on fluctuating skeletal muscle weakness and at least one of the following criteria: (1) positive response in the neostigmine test; (2) indicative repeated nerve stimulation results (> 10% decrease after stimulation of the face, ulna, axillary, and accessory nerves); and (3) positive for serum AChR or muscle-specific tyrosine kinase antibodies.

### Clinical information and collection

The primary outcome assessed in this study was aggravation of MG symptoms within 30 days of COVID-19 vaccination, where aggravation of MG was defined as patient subjective reported MG symptoms exacerbation or a 2-point increase in daily life myasthenia gravis activity (MGADL) score relative to before vaccination. Demographic and clinical characteristics, drug data, and vaccination information were collected. Symptom stabilization before vaccination was defined as complete clinical remission of > 3 months or no fluctuation in symptoms for > 3 months during medication. Symptom instability was defined as symptoms fluctuating within 3 months before vaccination, or patient needed adjustment of their medication due to disease fluctuations within 3 months before vaccination.

The 106 patients volunteered to be vaccinated that aimed to achieve herd immunity. Thirty-five patients consulted with community doctors before being vaccinated; however, none had consultations about vaccination at our center. Eighty-six patients provided signed informed consent to authorize the use of their clinical information without identification. Twenty patients could not attend the hospital due to the epidemic, and the researchers confirmed that they wished to provide vaccine information by telephone, without taking formal informed consent. The study was approved by the Ethics Committee of the First Affiliated Hospital of Sun Yat-Sen University (IIT-2022-068).

Statistical analyses were performed with SPSS 25 software (SPSS Inc., Chicago, IL, USA). All continuous quantitative data are presented as mean and standard deviation (SD). Differences in measurement data were compared with *t*-tests and variance analyses. We used univariate analysis and logistic regression analysis to understand the association of MG type and disease stability with disease exacerbation after vaccination. A *P* value of less than 0.05 was used to indicate statistical significance.

## Results

### Clinical characteristics of participants

Of a total of 1209 MG patients followed up during the investigation period, 106 were vaccinated against COVID-19 and were included in the study. The baseline characteristics of the 106 study participants were as follows: 38 (35.8%) were men, mean age was 42.3 years, mean age of onset was 33.6 years, mean course was 8.7 years, 53 (50.0%) had ocular MG, and all patients were classified I–II according to Myasthenia Gravis Foundation of America (MGFA) criteria (Table 1). AChR-Ab was positive in 74 (69.8%) patients and 21 (19.8%) patients had accompanying thymoma.

**Table 1:** Baseline characteristics of patients with MG vaccinated against COVID-19

Variable	Number (%) (total *n* = 106)
Sex *n* (%)	
Male	38 (35.8)
Female	68 (64.2)
Age (years, mean (SD))	42.4 (14.1)
Age at onset, mean (SD)	33.6 (17.7)
Disease course (years), mean (SD)	8.7 (8.2)
MG type, *n* (%)	
Ocular MG	53 (50.0)
Generalized MG	53 (50.0)
AChR-Ab, *n* (%)	
Positive	74 (69.8)
Negative	19 (17.9)
NA	13 (12.3)
Thymectomy, *n* (%)	
Yes	28 (26.4)
No	78 (73.6)
Thymoma, *n* (%)	
Yes	21 (19.8)
No	85 (80.2)
Disease status before vaccination *n* (%)	
Stable	102 (96.2)
Unstable	4 (3.8)
Duration of stability of MG at vaccination (months, mean (SD))	19.0 (20.1)
COVID-19 vaccine type, *n* (%)	
Inactivated vaccine	102 (96.2)
Adenoviral vector vaccine	2 (1.9)
Recombinant protein vaccine	2 (1.9)
Fully vaccinated, *n* (%)	
Incomplete	21 (19.8)
Complete	85 (80.2)
Booster vaccination	2 (1.9)
Worsening of MG symptoms, *n* (%)	
Yes	10 (9.4)
No	96 (90.6)
Adverse vaccine reactions, *n* (%)	
No	94 (88.7)
Pain at the inoculation site	6 (5.7)
Tired	5 (4.7)
Dizzy	1 (0.9)

*COVID-19*, coronavirus disease 2019; *AChR-Ab*, acetylcholine receptor antibody; *MG*, myasthenia gravis; *NA*, not available; *IQR*, interquartile range

#### Generalized information on vaccination in MG patients

At the time of vaccination, 21 (19.8%) patients were in complete clinical remission, and 85 (80.2%) patients were taking medication (Table 2). Furthermore, 102 (96.2%) patients acquired symptom stabilization before vaccination, with median duration of stable condition of 12 months; 4 (3.8%) patients had unstable symptoms. The majority of patients (*n* = 102) were vaccinated with inactivated virus vaccine; two patients received the adenovirus vector vaccine, and two the recombinant protein vaccine. A total of 85 patients had completed the whole inoculation course, two of whom had received booster doses, while 21 people had only received a single dose. “Stable” was defined as complete clinical remission lasting more than 3 months or stable symptoms during medication, without fluctuation.

**Table 2:** Status of patients vaccinated against COVID-19 while using drugs to treat MG

Medical treatment during vaccination	Number (%) (total *n* = 106)
No	21 (19.8)
Yes	85 (80.2)
Pyridostigmine bromide	79 (74.5)
Immunosuppressants	52 (49.1)
Glucocorticoids alone	25 (23.6)
Glucocorticoids + azathioprine	13 (12.3)
Glucocorticoids + leflunomide	6 (5.7)
Glucocorticoids + tacrolimus	2 (1.9)
Glucocorticoids + azathioprine + leflunomide	1 (0.9)
Glucocorticoids + azathioprine + tacrolimus	1 (0.9)
Azathioprine + leflunomide	1 (0.9)
Leflunomide	1 (0.9)
Tacrolimus	1 (0.9)

#### Small part of MG patients with worsening symptoms after vaccination

A total of 10 (9.4%) patients presented with worsening symptoms after vaccination (Table 3), of whom 8 presented with slight symptom worsening, which resolved rapidly. Two patients presented with serious worsening of symptoms, such as difficulty swallowing, labored breathing, and weak limbs, which required inpatient treatment. Furthermore, 12 participants had other common adverse reactions after vaccination: 6 felt pain at the vaccination site, 5 felt tired, and 1 felt dizzy.

**Table 3:** Univariate analysis of patients with worse MG condition after vaccination

Variable	Patients whose MG did not worsen (*n* = 96)	Patients with aggravated MG symptoms (*n* = 10)	*X*^2^	*P*
MG type, *n* (%)				
Ocular MG	51 (53.1)	2 (20.0)	3.975	0.046
Generalized MG	45 (46.9)	8 (80.0)		
MG disease status before vaccination, *n* (%)				
Stable	94 (97.9)	8 (80.0)		0.044
Unstable	2 (2.1)	2 (20.0)		

*MG*, myasthenia gravis; *P* < 0.05 was considered statistically significant. The minimum expected frequency in the chi-square test of whether MG was stable before vaccination was 0.38, and Fisher’s exact test was used

Univariate analysis indicated that generalized MG and unstable status before vaccination were associated with worsened muscle weakness (Table 3). Furthermore, a logistic regression model detected a significant association between elevated risk of increased muscle weakness after vaccination and unstable disease status before vaccination (Table 4).

**Table 4:** Logistic regression analysis of risk factors associated with aggravation of MG after vaccination

Variable	OR (95% CI)	*P*
Disease status before vaccination		
Stable	1.00	
Unstable	15.564 (1.524–156.904)	0.021

*MG*, myasthenia gravis; *P* < 0.05 was considered statistically significant. *OR*, odds ratio. *P* < 0.05 was considered statistically significant

All patients were vaccinated with inactivated vaccine, two had unstable disease at the time of vaccination, the median duration of muscle weakness exacerbation was 4.5 days, and the mean duration from vaccination to symptom worsening was 7.4 days. Two patients required hospitalization (cases 1 and 9) and three patients needed adjustments to their treatment plan (two patients taking glucocorticoids and one patient taking pyridostigmine bromide improved), while the remaining five patients did not need changes to their original treatment plan, and their symptoms resolved within a few days (Table 5). The change in the MGADL score of patients with aggravation after COVID-19 vaccination is shown in the Supplementary Part (Table 6).

**Table 5:** Clinical characteristics of patients with aggravation of MG after COVID-19 vaccination

ID	Sex	Age (years)	Age at onset (years)	Disease course (years)	MG type	AChR-Ab	Thymoma	PYB	Steroid	Other IS therapy	Stable or not	Stable duration (months)	Time of MG aggravation (days after vaccination)	Worsened symptoms	Therapy plan
1	F	30	26	4	GMG	+	Yes	Yes	No	Yes	Yes	36	30	Weak swallowing and breathing	Hospitalization
2	M	43	37	6	GMG	+	No	Yes	No	No	Yes	48	2	Weakness of limbs	Resolved within 2 days
3	F	61	60	1	GMG	+	No	Yes	No	No	Yes	5	10	Ptosis	Resolved within 7 days
4	F	33	23	10	GMG	+	No	Yes	No	No	Yes	60	10	Diplopia	Methylprednisolone 4 mg/d Resolved within 7 days
5	F	36	35	1	GMG	—	No	No	No	No	Yes	5	2	Ptosis, diplopia	resolved within 7 days
6	M	40	38	2	GMG	+	No	Yes	Yes	No	No	0	7	Ptosis, diplopia	Prednisone 5 mg/d Resolved within 7 days
7	F	43	15	28	OMG	+	No	Yes	Yes	Yes	Yes	24	2	Diplopia, limb weakness	Resolved within 9 days
8	F	32	3	29	OMG	+	Yes	Yes	Yes	No	Yes	5	2	Ptosis, limb weakness	Resolved within 5 days
9	F	38	38	1	GMG	+	Yes	No	No	No	Yes	9	7	Weak chewing, swallowing, breathing	Hospitalization
10	M	33	30	3	GMG	NA	No	No	No	No	No	0	2	Weakness of limb and chewing	PYB 60 mg TID, resolved within 7 days

*ID*, identification number; *MG*, myasthenia gravis; *F*, female; *M*, male; *AChR-Ab*, acetylcholine receptor antibody; *PYB*, pyridostigmine bromide; *IS*, immunosuppressants; *TID*, three times daily; *GMG*, generalized MG; *OMG*, ocular MG

## Discussion

For patients with MG, the most common factor influencing symptom deterioration is infection, and the global COVID-19 pandemic placed many patients with MG and immunosuppressed status at risk of infection. A French study reported that 34 patients with MG were infected with COVID-19, of whom 28 (82%) recovered and 5 (15%) died [7]. An observational study in the Czech Republic suggested that, of 93 patients with MG infected with COVID-19, 35 (38%) developed severe pneumonia after infection, and 10 (11%) died of COVID-19 infection [8]. Although there have been no large global research studies that have described the condition and prognosis of patients with MG infected with SARS-CoV-2, the findings described above indicate that such patients are more prone to severe pneumonia and myasthenia crisis, as well as having higher mortality rates. Deterioration of patients with MG during COVID-19 infection is associated with previous long-term high-dose corticosteroid treatment, older age, thymoma, and more recent treatment with rituximab [8]. Therefore, it is particularly important for patients with MG to be vaccinated against COVID-19 to protect them from the infection.

Theoretically, vaccines may trigger or exacerbate autoimmune diseases [9]; therefore, it is reasonable to question the safety of the COVID-19 vaccine in patients with MG. Several previous studies have evaluated the safety of various vaccines in patients with autoimmune diseases; however, there is no strong evidence that vaccination is associated with the exacerbation of autoimmune disease [10]. Strijbos et al. conducted a study of 47 patients with MG and serum positivity for AChR antibody, who received an influenza vaccine or placebo, and found no evidence that the influenza vaccine aggravated symptoms in these patients [11]. Furthermore, a multicenter observational study in South Korea found that influenza infection was a specific and important risk factor for increased MG symptoms, rather than influenza vaccination; 10 (40%) patients had worse MG symptoms after influenza, while only 2 (1.5%) had worse MG symptoms after influenza vaccination. Furthermore, the rate of aggravation of symptoms in patients with influenza (10/25, 40%) was significantly higher than in those with common cold (15/96, 15.6%; *p* = 0.006) [12]. Therefore, it is very important to prevent influenza infection in patients with MG. Previous studies showed that vaccination did not exacerbate the autoimmune diseases, systemic lupus erythematosus, dermatomyositis/polymyositis, and Sjogren’s syndrome [13]. Studies of vaccination against COVID-19 in patients with multiple sclerosis (MS) demonstrated that RNA, DNA, protein, and inactivated virus vaccines are likely safe for patients with MS. In addition, although viral vector vaccines were associated with a few central demyelinating events, their benefits may outweigh the risks if there is no alternative, and live-attenuated vaccines should be avoided in patients receiving DMT drug therapy [14].

All 106 patients with MG included in this study were classified with I to II according to the MGFA criteria, including 21 patients in complete clinical remission and 39 who were receiving low-dose glucocorticoids (equivalent to 2.5–10 mg of prednisone). A total of 27 patients were taking immunosuppressive agents, including tacrolimus, azathioprine, and leflunomide. Our findings showed that, in the majority of patients with MG vaccinated against COVID-19, the vaccines were safe and showed no aggravation of myasthenia symptoms after vaccination. A total of 10 patients (9.4%) had worse muscle weakness, 8 showed slight deterioration, 5 resolved quickly within a few days without medication adjustment, and 3 patients resolved within days, after adjustment of medication (2 with the addition of low-dose steroid and one with addition of pyridostigmine bromide). Two of the 10 cases with aggravation presented with weakness affecting swallowing and breathing and required hospitalization; these two cases were young women with generalized MG (GMG) and thymoma, but did not reach MG crisis or need mechanical ventilation. This finding suggests that the immune status of patients with GMG and thymoma differs from that of other MG patients, and requires full evaluation before vaccination.

Twelve patients presented with common adverse vaccine reactions identical to those observed in the general population, all symptoms of which disappeared after a few days without intervention, suggesting that the COVID-19 vaccine is safe for patients with stable MG. None of the vaccinated patients with MG included in this study has been infected with COVID-19 to date. A domestic observational study of COVID-19 vaccination in patients with MG in 2021 [15] also supported our conclusion, reporting two cases of mildly worsened weakness among 22 patients with MG following COVID-19 vaccination.

There was also a case report of a 5-year-old patient with MG who was vaccinated against COVID-19 with the Moderna vaccine and presented with MG crisis [16]. It can be inferred from our findings that the inactivated virus vaccine may be safe for patients with MG, while further studies on the safety of other mRNA, DNA, and viral vector COVID-19 vaccines are needed. Previous studies have not reported any association between symptom exacerbation after vaccination and disease status before vaccination [17], although it has been suggested that patients with MG vaccinated against COVID-19 may produce a COVID-MG-associated cytokine storm that could further aggravate the symptoms of muscle weakness [18]. Whether this risk is higher in patients with GMG combined with thymoma requires further research. Ideally, patients with MG should be vaccinated when their symptoms are stable; Tackenberg et al. proposed that symptoms should be stable for more than 4 months [19], while Strijbos et al. stated that stable MG symptoms should have been stabilized for more than 3 months before vaccination is recommended [11]. General adverse reactions to the vaccine in patients with MG, such as pain and fatigue, occurred in 11% of the vaccinated population, similar to rates in healthy people. We cannot exclude that the aggravation of muscle weakness symptoms after vaccination observed in ten patients in this study was not a directly related phenomenon; that is, the aggravation of the disease during that specific period may not have been directly related to the vaccine. In our study, we found that patients with GMG or with unstable condition before vaccination were significantly more likely to experience aggravated muscle weakness after vaccination than those with stable condition, as two cases among four patients with unstable condition presented with muscle weakness after vaccination.

Vaccine efficacy is another important issue in immunocompromised patients. Immunosuppressive drugs, such as rituximab and methotrexate, can reduce humoral responses and inhibit the production of neutralizing antibodies [20, 21]. Most patients with MG require years, or even lifelong immunosuppressive therapy, including corticosteroids and non-steroidal immunosuppressive drugs, and it has been suggested that only cell-depleting medication (eculizumab, rituximab, etc.) and sphingosine-1-phosphate modulators may attenuate the vaccine response. Whether patients with MG can establish a normal serum anti-COVID-19 antibody titer after receiving immunotherapy remains to be determined; however, in epidemic situations, even lower levels of antibodies are better than no vaccination.

## Conclusion

Due to the lack of randomized controlled trials of anti-COVID-19 vaccine in patients with MG, adverse events leading to aggravation of MG require further study, and additional clinical studies are needed to determine the prevalence of such adverse outcomes. Our results suggest that inactivated vaccine of COVID-19 is relatively safe for patients with stable disease status, but that vaccination should be postponed for patients with unstable GMG, particularly those with thymoma. Patients with MG should take appropriate treatment to achieve stable disease status before vaccination, based on relative immune homeostasis, to receive vaccine protection during the COVID-19 pandemic.

## Supplementary information

ESM 1

ESM 2
