# Supplementary material for: An observational study on the safety of COVID-19 vaccination in patients with myasthenia gravis
Source: Neurol Sci. 2023 May 9;44(7):2239–45. doi: 10.1007/s10072-023-06811-y (PMC10166684; doi:10.1007/s10072-023-06811-y)
Supplement: Supplementary file 1 — ESM 1 [file 10072_2023_6811_MOESM1_ESM.doc]

**Table 6 Daily life myasthenia gravis activity change of patients with aggravation of MG after COVID-19 vaccination**

| ID | 1 | 2 | 3 | 4 | 5 | 6 | 7 | 8 | 9 | 10 |  |
| --- | --- | --- | --- | --- | --- | --- | --- | --- | --- | --- | --- |
| Pre-MGADL | 2 | 2 | 1 | 1 | 2 | 3 | 2 | 1 | 3 | 3 |  |
| Post-MGADL | 10 | 4 | 4 | 5 | 6 | 5 | 6 | 5 | 8 | 5 |  |
|  | | | | | | | | | | | |

Abbreviations: ID, identification number; MGADL: Daily life myasthenia gravis-activity of daily living profile; Pre: before vaccination,

Post: after vaccination.
