# Supplementary material for: An observational study on the safety of COVID-19 vaccination in patients with myasthenia gravis
Source: Neurol Sci. 2023 May 9;44(7):2239–45. doi: 10.1007/s10072-023-06811-y (PMC10166684; doi:10.1007/s10072-023-06811-y)
Supplement: Supplementary file 2 — ESM 2 [file 10072_2023_6811_MOESM2_ESM.pdf]

中山大學附属第一医院
THE FIRST AFFILIATED HOSPITAL,SUN YAT-SEN UNIVERSITY
地 址:广州市中山二路58号
电 话:总机87755766 传真:87750632

August 26th , 2022

Statement for ethical approval in the study of Dr.Liu Weibin, et al.

The design and performance of the research project, which was conducted by Dr.Liu Weibin, et al. about "An observational study on the safety of COVID-19 vaccine in patients with myasthenia gravis" (Application ID:[2022]113), conformed to the ethical standards of Helsinki Declaration and our national legislation. This project was approved by the Medical Ethical committee for Clinical Research and Animal Trials of the First Affiliated Hospital of Sun Yat-sen University.

Yours sincerely,

Churong Yan

Churong Yan, MD.
Chairman
Medical Ethical Committee
The First Affiliated Hospital
Sun Yat-sen University

中山大学附属第一医院临床科研和实验动物伦理委员会
